# Acute and Subchronic Toxicological Study of the Cocktail Extract from *Curcuma xanthorrhiza* Roxb, *Phyllanthus niruri* L. and *Morinda citrifolia* L.

**DOI:** 10.1155/2024/9445226

**Published:** 2024-03-27

**Authors:** Idah Rosidah, Tiya Novlita Renggani, Nisrina Firdausi, Sri Ningsih, Prasetyawan Yunianto, Devi Permatasari, Olivia Bunga Pongtuluran, Hismiaty Bahua, Julham Efendi, Siska Andrina Kusumastuti, Nuralih, Sjaikhurrizal El Muttaqien, Nizar, Susi Kusumaningrum, Kurnia Agustini

**Affiliations:** ^1^Research Center for Pharmaceutical Ingredients and Traditional Medicine, National Research and Innovation Agency (BRIN), Bogor, Indonesia; ^2^Research Center for Agroindustry, National Research and Innovation Agency (BRIN), Bogor, Indonesia; ^3^Research Center for Sustainable Production System and Life Cycle Assessment, National Research and Innovation Agency (BRIN), Tangerang Selatan, Indonesia; ^4^Research Center for Vaccine and Drugs, National Research and Innovation Agency (BRIN), Bogor, Indonesia; ^5^Directorate of Utilization of Research and Innovation by Industry, National Research and Innovation Agency (BRIN), Jakarta, Indonesia

## Abstract

*Curcuma xanthorrhiza* Roxb*, Phyllanthus niruri* L., and *Morinda citrifolia* L. are Indonesian medicinal herbs used empirically as traditional therapeutics for maintaining health. The cocktail extract of these three plants (CECPM) had been developed and demonstrated immunostimulant activity in rats. This study aimed to evaluate the acute and subchronic toxicity of CECPM in vivo. The acute toxicity assay was conducted by orally administering a range dose of CECPM (313, 625, 1250, 2500, or 5000 mg/kg body weight (bw) on female mice once and then evaluating the toxic symptom every day for 14 days later. The chronic toxicity test was carried out by giving various doses of CECPM (600, 800, and 1000 mg/kg·bw) to female and male rats orally continuously for 90 consecutive days. The signs of toxicities were evaluated at the 90- and 28 days postadministration. The acute oral toxicity assays showed that there was no toxic syndrome and mortality found during the period of the experiment. The lethal dose level (LD_50_) of CECPM was more than 5000 g/kg, which was categorized as practically non-toxic. Meanwhile, in the sub-chronic toxicity study, some parameters tested at 90 days postadministration and after 28 days of withdrawal, such as the body weight, relative organ weight, food intake, hematological and biochemical blood parameters, and also histopathological examination of five primary tissues (heart, liver, kidney, spleen, and lung) revealed no abnormalities. There was no-observed adverse effect level (NOAEL) for the present study of CECPM 1000 mg/kg·bw of the rat. Therefore, it is concluded that the orally administered CECPM was relatively nontoxic during acute and subchronic toxicology studies.

## 1. Introduction

Traditional medicine derived from natural products has a rich history and remains extensively utilized in numerous countries for upholding health, as well as preventing and treating various illnesses [1, 2]. Traditional medicine practices are typically passed down through generations, originating from ancestors and continuing as part of a cultural heritage. Consequently, the general population tends to consume these remedies without apprehension regarding their potential toxicity [3]. As the use of medicinal plants and their products rises, there is a growing concern about the potential toxicology associated with herbal medicine. Consequently, assessing the toxicity of medicinal plants utilized in folk medicine becomes crucial to ensuring the safety of human beings.

Natural product preparation can be composed using a single or poly-extract (polyherbal or herbal formulation). The term polyherbal formulations refers to those pharmaceutical preparations that consists of more than one herb as a component for increased therapeutic effectiveness. Traditionally, polyherbal formulations were employed with the dual purpose of not just restoring a healthy body but also preventing the recurrence of ailments. Another benefit using combination herbs in proportioned rations was to reduce the toxicity associated with individual herbs, consequently enhancing their therapeutic effects [4].

*C. xanthorrhiza* Roxb, *P. niruri* L., and *M. citrifolia* L are known as empirical traditional herbs for treating various diseases in many ancient herbal formulas. *C. xanthorrhiza* Roxb is a native Indonesian plant known as Java turmeric or temulawak, belongs to the family Zingiberaceae. *C. xanthorrhiza* has been the major group of secondary metabolites, terpenoids, and curcuminoids [5]. *C. xanthorrhiza* contains a variety of terpenoids, such as xanthorrhizol is a bisabolene sesquiterpenoids compounds [6]. Curcuminoids are phenolic compounds that include curcumin, monodemethoxycurcumin, and bisdemethoxycurcumin [7, 8]. According to previous research, the rhizome has shown the effects of antioxidant, antibacterial, anti-inflammatory, anticancer, antidiabetes, and hepatoprotector [9–15]. In addition, researchers have reported the safety study of *C. xanthorrhiza* on animals. The aqueous extract of *C. xanthorrhiza* up to a dose of 2 g/kg·bw orally did not demonstrate any signs of toxicity in mice or rats [16]. The ethanolic extract of *C. xanthorrhiza* contained 0.1238 mg of xanthorrhizol each mg exhibited no toxic effect in mice until a dose of 5 g/kg·bw [17]. Rats administered with the major compound curcumin at the range dose of 1–5 g/kg·bw did not change the appearance, behaviour, and relative organ weights with the reported LD_50_ value being more than 2.0 g/kg·bw [18, 19]. The dosage form containing *C. xanthorriza* extract claimed as a hepatoprotector agent showed an LD_50_ more than 5 g/kg·bw and was categorized practically nontoxic [20].

*P. niruri*, family Euphorbiaceae, known as Meniran in Indonesia, has been widely used for traditional medicine. These medicinal plants contain several phytochemical constituents such as alkaloids, anthocyanins, chlorogenic acids, coumarins, flavonoids, lignans, phenolic acid, saponins, tannins, and terpenoids [21, 22]. Various studies showed that P. niruri extract have numerous biological activities, including antimicrobe, antiviral, antioxidative, antidiabetic, anti-inflammatory, and hepatoprotective activity [23–31]. Toxicity studies of *P. nirurri* had been conducted previously. It was reported that the LD_50_ value of the aqueous leaf extract of *P. nirurri* on mice and rats was more than 5000 mg/kg·bw, and it was classified as a nontoxic level [32, 33]. The lethal dose of the aqueous extract of *P. niruri* was recorded at 2590 mg/kg·bw in Swiss albino mice [34]. Similarly, another study involving *P. niruri* extract (without any information about the type of solvent) found the LD_50_ value to be higher than 1588 mg/kg·bw in Balb/c mice [35]. In a separate investigation, the ethanol extract of *P. niruri* demonstrated neither cytotoxic nor genotoxic effects, exhibiting nontoxicity on subchronic administration up to 300 mg/kg·bw in Sprague–Dawley rats [36]. In addition, the aqueous extract of *P. niruri*, administered at doses of 50, 150, or 250 mg/kg for 30 days, showed no genotoxic effects [37]. *Corilagin, an ellagitannin, is one of the major bioactive compounds in various plants include P. niruri.* Corilagin is safe and nontoxic even at higher dosages with LD_50_ 3500–5000 mg/kg in mice. Moreover, corilagin at dose 1000 mg/kg for four weeks did not show any adverse effect on body weight and behavior of the mice [38].

*M. citrifolia* or noni belongs to familiy Rubiaceae, commonly known as Mengkudu or Pace in Indonesia. *M. citrifolia* fruits have been reported to contain various compounds with activity, such as alkaloids, flavonoids, tannins, saponins, terpenoids, and glycosides [39, 40]. A number of major components have been identified in *M. citrifolia* plants, including scopoletin, octanoic acid, potassium, vitamin C, terpenoids, alkaloids, and anthraquinones [41]. *M. citrifolia* has been reported to have biological activities such as anti-inflammatory, antioxidant and immunostimulant, antifungal, anticancer, and antihypertensive [42–47]. Previous studies have already investigated the toxicity of this plant. Treatment Sprague–Dawley male rats with aqueous extract of *M. citrifolia* fruit did not reveal any signs of toxicity with the LD_50_ exceeding 3 g/kg·bw in rats [48]. Radhakrishnan and team reported that oral administration of *M. citrifolia* fruit ethanol extracts at 2 g/kg·bw to adult male and female Wistar albino rats did not result in any deaths or acute adverse effects based on clinical and mortality observations [49]. Moreover, sub-chronic oral administration of *M. citrifolia* water extracts at doses ranging from 0.25 to 1.0 g/kg·bw for six weeks did not exhibit any abnormalities in biochemical parameters [48]. However, chronic consumption of *M. citrifolia* fruit aqueous extract at doses equivalent to 2 mg/ml in drinking water (equivalent to 200 mg extract/kg in mice) for 6 months may lead to hepatotoxicity, weight loss, and eventual mortality. The hepatotoxic effect of the *M. citrifolia* fruit extract is unknown and may probably be due to the anthraquinon [50]. Abu et al. reported the Nordamnacanthal, Anthraquinone, isolated from the root of *M. citrifolia* L. shown to not be toxic at doses 10 mg/kg/day and 50 mg/kg/day of mice after administered orally for 28 days [51]. Damnacanthal from isolate of the root of *M. citrifolia* shown relatively low toxicity with a LD_50_ cut-off value of 2500 mg/kg [52].

Currently, we have developed polyherbal formulation that consists of these three plants extracts in a proportionated combination (termed with CECPM). Our *in vitro* preliminary studies showed that CECPM had immunostimulant activity in RAW 264.7. This formula demonstrated immunostimulant activity by stimulating cytokine proinflammatory such as IL-1*β*, IFN-*γ*, and TNF-*α* in RAW 264.7 cell. A cytotoxicity test on macrophage-like cell RAW 264.7 also demonstrated that CECPM did not show toxic effects up to 500 *μ*g/mL. All this immunostimulant activity data will be published in a separate report. Despite its potency, the safety of this polyherbal formulation at its effective dose needs to be evaluated which became the purpose of this study. Since CECPM is intended to be developed into a dosage form produced on an industrial scale, the safety evaluation methods adopted in this study are based on the Indonesian FDA guidelines.

## 2. Materials and Methods

### 2.1. Plant Materials and Preparation of CECPM

The plant materials were collected from different locations in Indonesia. The herbs of *P. niruri* and the rhizome of *C. xanthorrhiza* were obtained from Tawangmangu, Central Java, while the *M. citrifolia* fruit was received from Serpong, Banten. The rhizome and the fruit were cleaned and sliced, then dried in an oven to obtain less than 10% moisture level. The three materials were ground coarsely and mixed well with a certain ratio. The mixture was put in a closed percolation tank completed with a bag filter inside and streamed with 30% ethanol (CV. Budiarta, Indonesia) for 4 hours by the top circulation method. To generate a thick extract, the collected liquid extract was evaporated using a rotary evaporator at 150 bar and 45°C. The yield of CECPM was 6.65%, with curcumin content of 0.105 ± 0.02%.

### 2.2. Experimental Animals

Thirty BALB/c mice (30–40 g, female) were used for the 14-day acute toxicity test. Sixty female (140–220 g) and sixty male (160–290 g) rats were used for the 90-day subchronic toxicity test. The mice and rats were obtained from the national agency for drug and food control of the Republic of Indonesia (NADFC) or the Indonesian FDA (BPOM). The animals were maintained in well-ventilated cages and provided with rodent feed (Indofeed, Bogor, Indonesia) with access to water ad libitum. The animals were housed by their sex, each 5, in polycarbonate cages filled with hygiene and wood shaving in a controlled room (22 ± 3°C, 30%–70% humidity; day/night cycle of 12/12 hr). They were acclimatized for seven days before starting the experiment. The animal test was granted by the Ethics Committee of the Faculty of Medicine, University of Indonesia, with approval No. KET- 202/UN2.F1/ETIK/PPM.00.02/2022.

### 2.3. Experimental Design

#### 2.3.1. Acute Toxicity Study

The procedure for the toxicity study was carried out based on the guidelines for in vivo nonclinical toxicity from BPOM [53]. An acute toxicity study was conducted using the conventional median lethal dose (LD_50_) method. All the mice were fasted overnight from food before dosing with the extract. Thirty healthy BALB/c mice were randomly divided into one control group and five treatment groups (female, *n* = 5). The CECPM was dissolved in CMC 0.5% solution and administered orally to each mouse at five doses of 313, 625, 1250, 2500, and 5000 mg/kg·bw. They were monitored for the first 4 hours, 24 hours, and then for 14 days for any toxic signs and mortality. The changes in general behaviour and mortality of the mice were observed and recorded. Moreover, the toxic symptoms and signs (i.e., tremors, convulsions, salivation, diarrhoea, lethargy, and coma) were monitored for 14 consecutive days. After overnight fasting, on day 15, the mice were sacrificed after exposure to a ketamine overdose and then cervical dislocation. Five organs such as liver, kidney, heart, spleen, and lung were isolated, cleaned, and weighed. Each animal's relative organ weight (ROW) was calculated by organ weight/corresponding bw.

#### 2.3.2. Subchronic Toxicity Study

A subchronic toxicity study was performed according to BPOM [53]. Sixty female and male rats were randomized into six groups, each ten male and ten female rats, namely, the control group (CMC 0.5%), three treatment groups (600, 800, and 1000 mg/kg·bw of CECPM), the satellite of control (CMC 0.5%), and the satellite of CECPM high dose (1000 mg/kg·bw) orally for 90 days. The satellite groups were kept for another 28 days without any CECPM treatment to assess reversibility effects. The CECPM was suspended in CMC 0.5% solution and administered once daily for 90 days. The daily observation focused on changes in general behaviour. The rat's body weight and feed supply were measured twice a week. At the end of the drug administration period, all rats were sacrificed, and then hematological blood, biochemical serum, ROW, and histopathological tissue analysis were performed.

*(1) Biochemical and Hematological Analysis*. At the end of the experimental period, all rats were fasted overnight with free access to water and then anaesthetized with ketamine. Blood samples were collected from each rat through orbital sinus puncture into EDTA-coated tubes. Hematological parameters were analyzed by an automatic hematology analyzer (*Sysmex XS800i*) including red blood cell (RBC), hemoglobin (Hb), hematocrit (Hct), the mean cell volume (MCV), mean cell hemoglobin (MCH), the mean cell hemoglobin concentration (MCHC), white blood cell (WBC), platelet, neutrophil, lymphocyte, monocyte, and eosinophil. The blood sample in non-EDTA tubes was collected and centrifuged at 3000 rpm for 10 min at 4°C (stored at −20°C) to obtain serum samples. The serum biochemical parameter analysis of glucose, cholesterol, triglycerides, urea, creatinine, enzyme aspartate transaminase (AST), and alanine transaminase (ALT) levels was done using spectrophotometry with a standard kit (Diasys, GmbH, Ltd).

*(2) Histopathological Examination of Organs*. Rats were euthanized and sacrificed for isolating five organs: liver, kidney, spleen, heart, and lung. Organs were weighed and fixed with a 10% buffered formalin solution. All tissues were dehydrated, embedded in paraffin, sectioned, and then stained with hematoxylin-eosin. Histopathological alterations were observed by an expert pathologist using a light microscope (Zeiss, Germany).

### 2.4. Statistical Analysis

All experimental data were expressed as the mean ± standard deviation (SD). A one-way ANOVA test or the Kruskal–Wallis test was performed to analyse all parameters, followed by Dunnett's post hoc test or Mann–Whitney test for comparisons between CECPM-treated groups and the control. A *P* value of <0.05 was considered statistically significant. The female and male rats, as well as the treatment and satellite groups, were separately evaluated.

## 3. Results

### 3.1. Acute Toxicity Study

Table 1 shows the effects of CECPM in mice after acute oral administration. There were no signs of toxicity and mortality at all levels of doses of CECPM administration (313, 625, 1250, 2500, and 5000 mg/kg·bw) for 14 days. There were no toxic symptoms in general behavioural effects, such as tremors, convulsions, salivation, diarrhoea, lethargy, and coma. In addition, there were no statistically significant differences between control and CECPM-treated animals in the data, specifically in body weights, organ weights, and relative organ weights for 14 days after treatment. The result of the acute toxicity study indicated that the LD_50_ of the CECPM was estimated to be above 5000 mg/kg·bw.

### 3.2. Subchronic Toxicity Study

#### 3.2.1. Clinical Signs and Mortality

The subchronic toxicity test was performed on male and female rats with repeated doses for 90 days of treatment with CECEPM and 28 days of recovery. The result demonstrated that no mortality and behaviour changes or signs of clinical toxicity were apparent in all the groups during the administration periods.

#### 3.2.2. Body Weight, Food Consumption, and Organ Weight

The subacute toxicity of CECPM at all the doses used did not produce any noticeable symptoms of toxicity or mortality in all the treated rats. During the treatment period, there were no significant variations in food intake, body weight, and organ weight between the treatment and control groups in female and male rats (Tables 2 and 3). In addition, there were no significant differences in the relative weights of the rats' hearts, liver, kidneys, and lungs among the different groups compared with the control group. However, the relative spleen weight of female and male rats significantly decreased in rats treated with 800 mg/kg·bw of CECPM. Meanwhile, there were significant differences in the relative weight of the spleen in rats treated with 800 mg/kg·bw in both sexes compared to control group, but no significant with 600 mg/kg and 1000 mg/kg·bw in the CECPM-treated group.

#### 3.2.3. Hematological Analysis

Hematological analyses were performed using blood plasma to evaluate possible changes in blood chemistry and detect possible changes related to the CECPM-treated compared to the control group. The result of the hematological analysis did not show significant variations in all doses compared to the control group in female and male rats presented in Table 4. The same results shown in the satellite group did not show any significant differences between the CECPM-treated groups and the control in all parameters.

#### 3.2.4. Serum Biochemical Analysis

**Figure 1:**
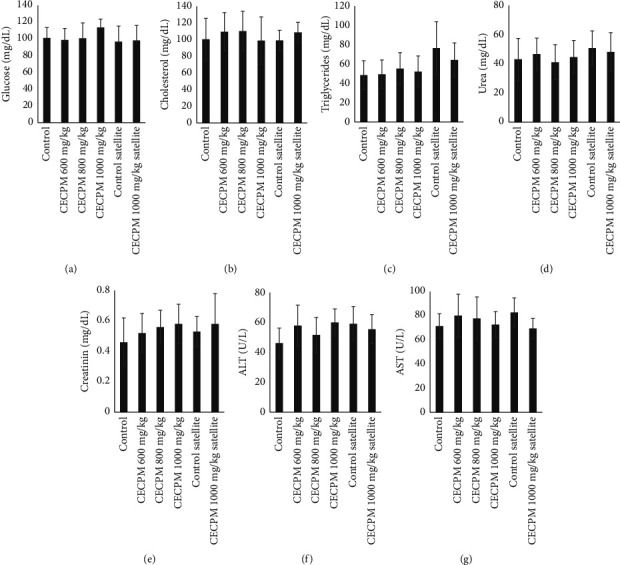
Effect of CECPM on biochemical profile in female rats in sub-chronic oral toxicity study. (a) Glucose, (b) cholesterol, (c) triglycerides, (d) urea, (e) creatinine, (f) ALT, and (g) AST. Data are expressed as means ± SD of ten animals in each group.

**Figure 2:**
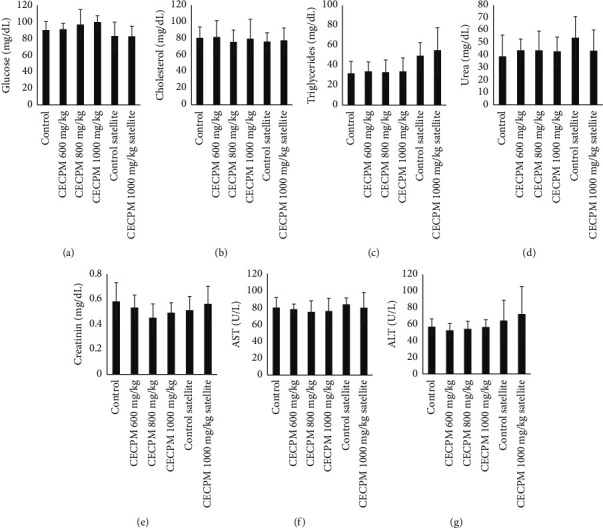
Effect of CECPM on biochemical profile in male rats in sub-chronic oral toxicity study. (a) Glucose, (b) cholesterol, (c) triglycerides, (d) urea, (e) creatinine, (f) ALT, and (g) AST. Data are expressed as means ± SD of ten animals in each group.

Repeat oral doses of CECPM administration for 90 days resulted in no statistically significant changes in glucose, cholesterol, triglycerides, urea, creatinine, AST, and ALT levels relative to the control group of each sex of rats (Figures 1 and 2). Similarly, the satellite group showed no significant differences in all biochemical parameters of 1000 mg/kg·bw of CECPM-treated compared to those of the control group in female and male groups.

#### 3.2.5. Histopathological Analysis

**Figure 3:**
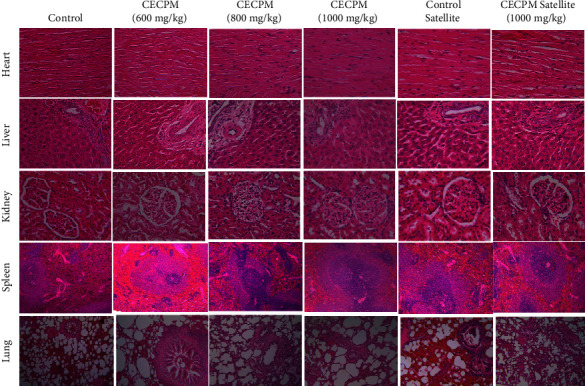
Effect of CECPM oral subchronic toxicity study on the heart, liver, kidney, spleen, and lung in histomorphology in female rats. Histological sections were visualized by staining with hematoxylin-eosin and observed by optical microscope with magnification ×40 (heart) and ×100 (liver, kidney, spleen, and lung). No significant (*P* > 0.05) alteration was observed in all treatment groups than the control group.

**Figure 4:**
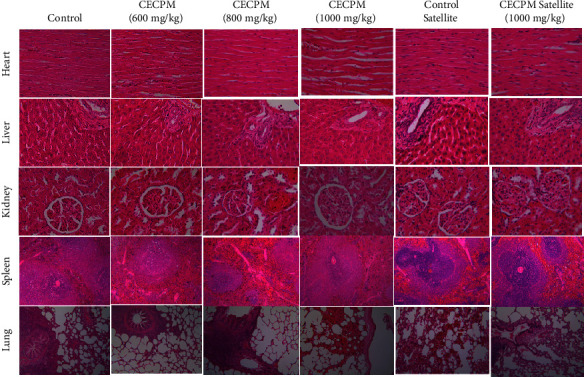
Effect of CECPM oral subchronic toxicity study on the heart, liver, kidney, spleen, and lung in histomorphology in male rats. Histological sections were visualized by staining with hematoxylin-eosin and observed by optical microscope with magnification ×40 (heart) and ×100 (liver, kidney, spleen, and lung). No significant (*P* > 0.05) alteration was observed in all treatment groups than the control group.

The histological sections of the heart, liver, kidney, spleen, and lung of the control and CECPM-treated rats are shown in Figures 3 and 4. Histopathological examination of all organs (heart, liver, kidney, spleen, and lung tissue) of female and male rats showed no significant pathological changes between control and treatment groups. Moreover, in the satellite group, there were no abnormalities in the rats received 1000 mg/kg·bw CECPM-treated after 28 days of withdrawal time.

## 4. Discussion

Toxicological studies have been used to evaluate the toxicity effects and mechanisms of action of drugs and chemicals [54]. Toxicological studies of medicinal plants are required to investigate the potential toxicity and ensure the safety of the drug candidate. Although many plant extracts have been traditionally used to treat various diseases, there are only a few experimental data on the safety of polyherbal or extract formulas. This study was carried out to explore the acute and subchronic toxicity of CECPM in animals. To the best of our knowledge, this is the first study to evaluate the toxicity of the cocktail extract containing three medicinal plants: *C. xanthorrhiza, P. niruri*, *and M. citrifolia.*

*C. xanthorriza* and *P. niruri* are native Indonesia medicinal plants that can be used as an immunostimulant. In addition, *M. citrifolia* is a *native* fruit commonly *found* in *tropical countries* originating from *Southeast Asia* (Indonesia) and Australia. *C. xanthoriza* has activity to modulate immune response including innate and adaptive immune systems. *C. xanthorriza* polysaccharide extract increased nitric oxide (NO) production and thrombocytes in a rat model-induced cyclophosphamide [12]. Methanol extract of *C. xanthorrhiza* could modulate leukocytes phagocytosis in human whole blood and increase NO, hydrogen peroxide (H_2_O_2_), TNF-*α*, and prostaglandin E2 (PGE2) production in RAW 264.7 cells [55, 56]. The ethyl acetate extract of *P. niruri* has increased the activity of the phagocytosis index and capacity macrophages of male mice induced by *Staphylococcus aureus* [57]. Hong et al. demonstrated that the standardized water extract of *M. citrifolia* stimulates macrophages, induces NO production, and upregulates the mRNA expression of cytokine genes by activating NF‐*κ*B and AP‐1 signalling pathways without any severe cytotoxicity. The water extract of *M. citrifolia* also stimulated splenocytes isolated from mice by inducing NO production and expression of immunostimulatory cytokines. Moreover, the water extract of *M. citrifolia* induced immunostimulant by modulating populations of splenic immune cells, especially by increasing the population of IFN‐*γ*+ NK cells [58]. Based on research by Nayak and Mengi, the potential of *M. citrifolia* as an immunostimulant has been elucidated. It works through a stimulation of phagocytic of neutrophils and mediates the release of IL-6 on in vitro phagocytosis of *Candida albicans* spores [59]. Palu et al. (2008) demonstrated that *M. citrifolia* juice stimulated the cannabinoid receptors (CB2) decrease in interleukin-4 (IL-4) levels with a concomitant increase in interferon-*γ* (IFN-*γ*) levels [60]. Sagala and Murwanti report that the combinations of ethanol extracts of *P. niruri*, *T. flagelliforme*, and *P. crocatum* can improve nonspecific immune responses by increasing the phagocytic index and phagocytic capacity of macrophages peritoneum of mice [61].

Based on these previous reports, it can be concluded that the immunostimulatory effect of the CECPM formula may come from its extract component. The pharmacology property of *C. xanthorriza* are known to consist curcumin and polysaccharide [12, 62], and *M. citrifolia* reportedly contains polysaccharide, anthraquinone, alkaloid, and deacetylasperulosidic acid [58, 59, 63, 64]. In CECPM, we analyzed curcumin as a marker compound for extract standardization. Curcumin, a polyphenol compound, has been the subject of intensive investigations due to its various pharmacological activities [17].

In the present study, the oral acute toxicity evaluation showed that the LD_50_ value of CECPM was estimated to be above 5000 mg/kg·bw. The CECPM could be categorized as a nontoxic herbal drug class following the Hodge and Sterner classification [65]. The safety profile of CECPM in this study is also consistent with a previous study reporting the toxicity profile of a single extract of CECPM; the LD50 of *C. xanthorriza* and *P. nirurri* was more than 5000 mg/kg·bw, and the administration of *M. citrifolia* up to a dose of 2000 mg/kg did not cause any signs of toxicity and mortality [24, 38–42]. Similarly, the polyherbal combination containing each extract of CECPM, i.e., *Piper crocatum, Typhonium flagelliforme,* and *P. niruri* was safe up to 5000 mg/kg·bw on male and female Sprague*–*Dawley rats [66]. The median lethal dose of polyherbal contains *Andrographis paniculata, Boerhavia diffusa, Phyllanthus amarus,* and *Solanum nigrum* was found to be more than 2000 mg/kg·bw on Sprague–Dawley rats [67]. Acute toxicity testing of the extract combination of *P. niruri, Sonchus arvensis*, and *Nigella sativa* was hardly toxic to male and female Sprague–Dawley rats [68]. Moreover, the mixture of *C. xanthorriza* and Noni fruit extract has an LD_50_ of more than 2100 mg/kg·bw on male and female of the mice [69]. The combination of extracts of *C. xanthorrhiza* and *Gynura procumbens* comparison with the ratio 1 : 4 did not have the potential toxicity up to a dose of 2000 mg/kg in female Wistar strain rats [70]. The development of an anticancer formula containing three plant extracts, namely *Loratus* sp., *Selaginella tamariscina,* and *C. xanthorriza* was shown to be safe up to dose 3000 mg/kg·bw on Sprague–Dawley rats [71]. It is known that the administration of a mixture containing several medicinal plant extracts in a certain ratio to rats resulted in an additive (synergism) response. The factors of detoxification and kinetic interaction among the extract components are suggested as the main causes of the observed synergy [72]. The complex synergistic interactions among the herbs in complex formulations are believed to be able to enhance the bioavailability of active components, promote therapeutic effects, and/or reduce toxicity [73].

The toxicity of CECPM was evaluated by performing 90-day oral subchronic toxicity studies in male and female Sprague–Dawley rats. An investigation of the subchronic toxicity of plant-derived extracts on both male and female rats is needed due to distinct hormone responses. It is known that sex hormones can influence the response of the body to the tested natural products [74]. In addition, the inclusion of both male and female rats in this study may enhance the validity of the drug toxicity profile [75]. The subchronic toxicity study is used to detect toxic effects that may occur after administration of the repeated doses. The duration of substance administration to the animals should be no longer than 10% their maximum life span. This subchronic toxicity study is intended to gather information on the principal toxic effects that may suggest the target organs, the possibility of accumulation of drug substances, and an assessment of the NOAEL of exposure. This data can be used to select dose levels for chronic studies and establish safety criteria for human exposure [53, 76]. Three dose levels of 600, 800, and 1000 mg/kg·bw of CECPM were selected for the subchronic oral toxicity study in rats. The sub-chronic toxicity test was performed on male and female rats with repeated doses for 90 days of treatment and 28 days of recovery. The result demonstrated that no mortality and behaviour changes or signs of clinical toxicity were apparent in all the groups during the administration periods.

The mean body weights were measured to determine the effect of the CECPM on animal growth. The increase in body weight of all animals during the experimental period indicates that the CECPM did not have general toxic effects and reduction in appetite. Body weights are closely related to nutrition, water consumption, and stress factors in animals. The changes in body weight indicate adverse side effects after exposure to a substance [66]. The 10% change in the body weight may indicate a subchronic effect of the tested substance to the animal [77]. Based on results in Tables 2 and 3, there were no significant variations in food intake, body weight, and organ weight between the treatment and control groups in female and male rats. The relative weight of organ such as hearts, liver, kidneys, and lungs has not shown significant differences between the treated groups and the control (Tables 2 and 3). However, the relative spleen weight of female and male rats significantly decreased in rats treated with 800 mg/kg·bw of CECPM. Meanwhile, there were significant differences in the relative weight of the spleen in rats treated with 800 mg/kg·bw in both sexes compared to the control group, but no significant with 600 mg/kg and 1000 mg/kg·bw in the CECPM-treated group. The reduction of the relative spleen weight may be a minor finding, and it also exhibited no dose dependence without any treatment-related histological alteration. Therefore, these changes might be due to a normal biological variation.

Hematological data play a major role in determining the toxicity induced by drug substances, as blood is the main transport system for almost all metabolic processes in the body [78]. Thus, hematological data provided important information in preclinical and clinical studies, such as diagnosis evaluation, prognosis, assessment of the efficacy of therapy and toxicity of drugs and candidate drugs [79]. In the present study, hematological analyses were performed using blood plasma to evaluate possible changes in blood chemistry and detect possible changes related to the CECPM-treated compared to the control group. The result of the hematological analysis did not show significant variations in all doses compared to the control group in female and male rats. The same results shown in the satellite group did not show any significant differences between the CECPM-treated groups and the control in all parameters. Based on the results, the CECPM-treated rats up to 1000 mg/kg·bw (oral-90 days) are safe and nontoxic to all hematological parameters. Listyawati (2006) studied the chronic toxicity level of the ethanol extract of *C. xanthorrhiza* orally on mice's hematological and male reproduction systems. The results suggested that the orally administered C. xanthorrhiza at a dose of 150 mg/kg·bw did not induce a toxic effect on male mice's hematological characteristics and reproductive system after 90 days of treatment [80]. The powder of *M. citrifolia* fruit showed no adverse hematologic effects at the dose levels of 2000 and 5000 mg/kg·bw/day for 13 weeks [81]. The polyherbal containing *P. niruri* in rats also did not demonstrate significant differences in the hematological parameters between the treatment and control groups [66, 82].

Biochemical parameters play a significant role in monitoring the clinical symptoms induced by a toxin. [83]. Evaluation of kidney and liver function is important to assess toxicity profiling of plant extracts and ensure the survival of an organism [78, 84]. Renal and liver function tests may be used to detect the signs of toxicity induced by the plant extracts; the kidneys play an important role in the excretion of drug substances, and the liver is a major organ involved in the metabolism process of the drug substances [84]. Renal function tests could be evaluated by monitoring urea and creatinine levels, while liver function could be determined by measuring ALT and AST levels. The CECPM-treated group does not affect fasting blood glucose, cholesterol, and triglyceride levels in all rats, which are associated with cardiac function [85]. These findings were *in line* with the *study of* Murwanti 2023, which reported that the oral administration of polyherbal containing *P. niruri* extract for 90 days had no effect on the biochemical parameters in rats after the sub-chronic toxicity test [66]. Fatimah et al. found that the combination of extracted *P. niruri* and *Centella asiatica* on hematology in rats was normal and did not significantly change after 28 days of oral administration [82].

Histopathological assessments of organs may provide valuable evidence regarding the effects of a test chemical on their microscopic structures for the safety and toxicity assessment of medicinal plants [86]. This assessment is intended to investigate any abnormalities in the organ pathology [66]. The histopathological evaluation of five vital organs in female and male rats showed no significant pathological changes between control and treatment groups. Similarly, the satellite group showed normal and no microscopic changes occurred in the heart, liver, kidney, spleen, and the lung tissue in the rats receiving CECPM treatment after 28 days of withdrawal time. These results indicated that CECPM had a reversible effect on the microscopic examination. Thus, these findings suggest that administration of CECPM at doses up to 1000 mg/kg·bw once daily for 90 consecutive days has no toxic effect on the analyzed organs. A similar result was also obtained in the toxicity studies of polyherbal formulations in rats where no significant toxic effects were observed on the physical signs and symptoms, weight gain, food intake, hematological and biochemical parameters, and histopathology of organs [66, 87].

## 5. Conclusions

The present study reported that the acute oral toxicological studies of CECPM did not cause any mortality and toxicity effect at all levels of doses and LD_50_ greater than 5000 mg/kg·bw. Moreover, in a subchronic toxicological study, CECPM did not have a toxic potential effect at doses 600, 800, and 1000 mg/kg·bw in long-term treatment (90 days). Compared to the control group, there were no statistically significant treatment-related adverse effects on body weights, food consumption, hematology, and biochemistry parameters of animals. Similarly, there were no significant related histopathological changes in the CECPM-treated and control groups of animals. Hence, the NOAEL for long-term use of CECPM could be 1000 mg/kg·bw for both sexes. These results provide valuable valid data on the toxicity profile of CECPM. The safe toxicity profile is necessary for further development of CECPM as polyherbal formula of immunostimulant.

## Figures and Tables

**Table 1:** The body weight and relative organ weights of mice treated orally with CECPM for 14 days.

Parameters	Groups					
	Control	CECPM (313 mg/kg·bw)	CECPM (625 mg/kg·bw)	CECPM (1250 mg/kg·bw)	CECPM (2500 mg/kg·bw)	CECPM (5000 mg/kg·bw)
Body weight						
Initial weight (g)	36.68 ± 2.47	33.08 ± 3.51	36.04 ± 3.05	35.82 ± 3.72	36.24 ± 1.40	34.52 ± 2.89
Final weight (g)	38.90 ± 2.28	36.36 ± 3.17	38.76 ± 6.05	38.82 ± 4.01	39.80 ± 1.07	37.88 ± 2.61
Body weight gain (g)	2.22 ± 0.40	3.28 ± 4.46	2.72 ± 3.89	3.00 ± 1.49	3.56 ± 0.59	3.32 ± 1.16
Organ weight						
Kidney (g)	0.42 ± 0.03	0.43 ± 0.04	0.40 ± 0.04	0.44 ± 0.09	0.46 ± 0.02	0.43 ± 0.08
Liver (g)	2.06 ± 0.12	1.19 ± 0.19	1.92 ± 0.20	2.09 ± 0.23	2.04 ± 0.23	2.04 ± 0.23
Spleen (g)	0,16 ± 0.02	0.21 ± 0.03	0.20 ± 0.04	0.19 ± 0.05	0.23 ± 0.10	0.23 ± 0.10
Heart (g)	0.16 ± 0.02	0.16 ± 0.04	0.16 ± 0.03	0.16 ± 0.03	0.14 ± 0.01	0.14 ± 0.01
Lung (g)	0.23 ± 0.04	0.21 ± 0.05	0.22 ± 0.03	0.26 ± 0.07	0.26 ± 0.03	0.26 ± 0.03
Relative organ weight						
Kidney (%)	1.07 ± 0.10	1.22 ± 0.13	1.03 ± 0.09	1.14 ± 0.16	1.14 ± 0.19	1.14 ± 0.19
Liver (%)	5.30 ± 0.29	5.38 ± 0.42	4.99 ± 0.35	5.38 ± 0.25	5.40 ± 0.53	5.40 ± 0.53
Spleen (%)	0.41 ± 0.07	0.60 ± 0.07	0.51 ± 0.10	0.48 ± 0.11	0.61 ± 0.29	0.61 ± 0.29
Heart (%)	0.40 ± 0.04	0.44 ± 0.60	0.42 ± 0.06	0.41 ± 0.06	0.38 ± 0.03	0.38 ± 0.03
Lung (%)	0.59 ± 0.11	0.60 ± 0.13	0.59 ± 0.12	0.65 ± 0.11	0.67 ± 0.05	0.67 ± 0.05

Data are expressed as means ± SD of five animals in each group.

**Table 2:** Body weight, organ weights, and relative organ weights of female rats treated orally with CECPM for 90 days.

Parameters	Groups					
	Control	CECPM (600 mg/kg bw)	CECPM (800 mg/kg bw)	CECPM (1000 mg/kg bw)	Satellite	
					Control	CECPM (1000 mg/kg bw)
Body weight						
Initial weight (g)	181.62 ± 25.05	191.50 ± 25.53	185.52 ± 19.61	176.53 ± 22.65	195.08 ± 18.92	186.27 ± 16.92
Final weight (g)	226.33 ± 29.26	230.04 ± 22.03	226.69 ± 18.60	215.97 ± 27.26	245.27 ± 21.88	236.26 ± 16.70
Bogy weight gain (g)	44.71 ± 19.87	38.54 ± 13.06	41.17 ± 21.09	42.31 ± 16.00	50.19 ± 21.13	49.99 ± 15.46
Food intake (g)	20.68 ± 2.68	20.92 ± 1.77	20.35 ± 2.08	21.13 ± 2.67	21.25 ± 1.62	19.98 ± 1.77
Organ weight						
Kidney (g)	1.47 ± 0.20	1.55 ± 0.27	1.48 ± 0.19	1.39 ± 0.16	1.55 ± 0.20	1.49 ± 0.11
Liver (g)	6.14 ± 1.02	6.63 ± 0.94	6.25 ± 0.75	5.99 ± 0.76	6.50 ± 0.69	6.60 ± 0.71
Spleen (g)	0,50 ± 0.07	0.48 ± 0.08	0.43 ± 0.06	0.51 ± 0.13	0.49 ± 0.08	0.44 ± 0.08
Heart (g)	0.79 ± 0.09	0.79 ± 0.14	0.76 ± 0.09	0.78 ± 0.09	0.84 ± 0.09	0.82 ± 0.08
Lung (g)	2.31 ± 0.59	2.45 ± 0.63	2.18 ± 0.66	2.28 ± 0.81	2.27 ± 0.65	2.07 ± 0.81
Relative organ weight						
Kidney (%)	0.65 ± 0.03	0.67 ± 0.08	0.65 ± 0.04	0.65 ± 0.04	0.63 ± 0.07	0.63 ± 0.02
Liver (%)	2.71 ± 0.22	2.87 ± 0.17	2.75 ± 0.16	2.78 ± 0.14	2.66 ± 0.25	2.80 ± 0.24
Spleen (%)	0.22 ± 0.02	0.21 ± 0.03	0.19 ± 0.02^a^	0.24 ± 0.05	0.20 ± 0.03	0.18 ± 0.03
Heart (%)	0.35 ± 0.03	0.21 ± 0.03	0.34 ± 0.03	0.36 ± 0.03	0.34 ± 0.03	0.35 ± 0.04
Lung (%)	1.03 ± 0.30	1.07 ± 0.27	0.96 ± 0.26	1.05 ± 0.33	0.93 ± 0.27	0.87 ± 0.33

Data are expressed as the means ± SD of ten animals in each group. ^a^*P* < 0.05 compared to the control group.

**Table 3:** Body weight, organ weights, and relative organ weights of male rats treated orally with CECPM for 90 days.

Parameters	Groups					
	Control	CECPM (600 mg/kg bw)	CECPM (800 mg/kg bw)	CECPM (1000 mg/kg bw)	Satellite	
					Control	CECPM (1000 mg/kg bw)
Body weight						
Initial weight (g)	238.48 ± 23.93	249.43 ± 23.34	230.05 ± 37.29	240.30 ± 35.12	250.47 ± 19.35	235.70 ± 42.46
Final weight (g)	350.33 ± 42.92	339.27 ± 27.88	333.50 ± 29.34	341.06 ± 43.27	358.43 ± 34.22	365.22 ± 72.27
Body weight again (g)	111.85 ± 35.96	89.84 ± 30.99	103.45 ± 36.97	100.76 ± 36.11	107.96 ± 26.34	127.52 ± 84.31
Food intake (g)	26.56 ± 3.03	27.23 ± 3.41	21.10 ± 3.69	27.81 ± 3.39	28.04 ± 3.21	27.00 ± 2.90
Organ weight						
Kidney (g)	2.33 ± 0.23	2.14 ± 0.21	2.14 ± 0.20	2.28 ± 0.29	2.20 ± 0.24	2.28 ± 0.43
Liver (g)	9.48 ± 1.45	8.96 ± 0.97	8.92 ± 1.03	9.53 ± 2.31	8.55 ± 2.94	10.73 ± 2.67
Spleen (g)	0,76 ± 0.14	0.67 ± 0.20	0.56 ± 0.07	0.66 ± 0.11	0.59 ± 0.13	0.60 ± 0.13
Heart (g)	1.03 ± 0.16	1.03 ± 0.09	1.02 ± 0.10	1.04 ± 0.14	1.01 ± 0.12	1.03 ± 0.17
Lung (g)	2.48 ± 0.66	2.51 ± 0.78	2.85 ± 1.00	3.60 ± 2.75	3.68 ± 1.04	2.91 ± 0.72
Relative organ weight						
Kidney (%)	0.67 ± 0.03	0.63 ± 0.04	0.64 ± 0.03	0.67 ± 0.05	0.62 ± 0.05	0.63 ± 0.04
Liver (%)	2.70 ± 0.13	2.64 ± 0.09	2.67 ± 0.18	2.79 ± 0.51	2.39 ± 0.83	2.94 ± 0.24
Spleen (%)	0.22 ± 0.04	0.20 ± 0.04	0.17 ± 0.02^a^	0.20 ± 0.03	0.16 ± 0.03	0.17 ± 0.02
Heart (%)	0.29 ± 0.03	0.30 ± 0.02	0.31 ± 0.02	0.31 ± 0.02	0.28 ± 0.02	0.28 ± 0.02
Lung (%)	0.70 ± 0.14	0.74 ± 0.22	0.85 ± 1.00	1.08 ± 0.87	1.04 ± 0.32	0.82 ± 0.21

Data are expressed as the means ± SD of ten animals in each group. ^a^*P* < 0.05 compared to the control group.

**Table 4:** Effect of CECPM on hematological profile of rats in sub-chronic oral toxicity study.

Sex	Parameters	Groups					
		Control	CECPM (600 mg/kg·bw)	CECPM (800 mg/kg·bw)	CECPM (1000 mg/kg·bw)	Satellite	
						Control	CECPM (1000 mg/kg·bw)
Females♀	RBC (10^6^/*μ*L)	8.58 ± 0.55	8.56 ± 1.00	8.10 ± 1.86	8.76 ± 0.30	8.56 ± 0.63	8.68 ± 0.29
	HGB (g/dL)	15.77 ± 0.81	15.37 ± 1.55	14.53 ± 3.24	15.87 ± 0.54	15.6 2 ± 1.23	15.71 ± 0.48
	HCT (%)	44.76 ± 2.15	43.69 ± 4.32	44.44 ± 3.32	44.74 ± 1.15	44.34 ± 3.34	44.61 ± 0.94
	MCV (fL)	52.23 ± 1.90	51.16 ± 1.87	51.29 ± 2.48	51.07 ± 1.03	51.83 ± 0.97	51.43 ± 1.35
	MCH (pg)	18.40 ± 0.47	18.01 ± 0.52	17.97 ± 0.67	18.14 ± 0.26	18.24 ± 0.31	18.12 ± 0.42
	MCHC (g/dL)	35.25 ± 0.60	35.18 ± 0.42	35.06 ± 1.06	35.47 ± 0.53	35.23 ± 0.37	35.21 ± 0.39
	PLT (10^3^/*μ*L)	935.22 ± 160.81	1078.11 ± 217.48	839.43 ± 286.16	1022.67 ± 158.71	909.90 ± 317.85	934.70 ± 312.13
	WBC (10^3^/*μ*L)	9.93 ± 2.16	8.64 ± 2.82	7.10 ± 2.95	10.01 ± 3.09	10.74 ± 2.77	10.09 ± 1.57
	NEU (10^3^/*μ*L)	2.50 ± 1.07	2.76 ± 0.76	1.85 ± 1.25	2.41 ± 0.93	3.40 ± 0.86	3.30 ± 0.51
	LYM (10^3^/*μ*L)	6.18 ± 1.32	4.83 ± 1.97	4.43 ± 1.56	6.15 ± 1.64	5.90 ± 2.04	5.50 ± 1.17
	MONO (10^3^/*μ*L)	0.76 ± 0.36	0.70 ± 0.28	0.52 ± 0.37	0.69 ± 0.30	1.10 ± 0.33	0.96 ± 0.19
	EOS (10^3^/*μ*L)	0.35 ± 0.19	0.36 ± 0.22	0.19 ± 0.18	0.52 ± 0.39	0.35 ± 0.16	0.33 ± 0.17

Males♂	RBC (10^6^/*μ*L)	9.98 ± 0.35	9.57 ± 0.42	9.53 ± 0.94	9.81 ± 0.59	9.90 ± 0.93	10.11 ± 0.46
	HGB (g/dL)	16.55 ± 0.46	16.44 ± 0.85	16.53 ± 1.09	16.59 ± 0.99	16.36 ± 1.65	16.83 ± 0.58
	HCT (%)	47.48 ± 1.64	47.01 ± 2.23	47.26 ± 3.10	47.18 ± 2.76	46.43 ± 4.27	47.73 ± 1.84
	MCV (fL)	47.63 ± 1.99	50.16 ± 4.12	49.76 ± 2.68	48.12 ± 1.93	46.81 ± 0.94	47.29 ± 2.01
	MCH (pg)	16.60 ± 0.56	17.20 ± 0.62	17.40 ± 0.82	16.93 ± 0.82	16.52 ± 0.37	16.65 ± 0.48
	MCHC (g/dL)	34.87 ± 0.46	34.96 ± 0.42	35.04 ± 0.34	35.16 ± 0.50	35.30 ± 0.52	35.26 ± 0.60
	PLT (10^3^/*μ*L)	983.80 ± 179.22	874.10 ± 198.58	811.10 ± 306.07	760.33 ± 313.10	1040.25 ± 167.68	923.70 ± 325.93
	WBC (10^3^/*μ*L)	13.03 ± 2.45	11.39 ± 2.60	11.25 ± 1.76	11.74 ± 3.21	12.75 ± 3.14	14.09 ± 1.93
	NEU (10^3^/*μ*L)	2.70 ± 0.47	3.08 ± 0.49	3.29 ± 0.82	2.96 ± 0.81	3.20 ± 0.60	3.55 ± 0.81
	LYM (10^3^/*μ*L)	8.56 ± 2.12	7.65 ± 2.20	6.76 ± 1.21	7.10 ± 1.91	7.12 ± 1.67	7.97 ± 1.63
	MONO (10^3^/*μ*L)	1.06 ± 0.28	0.97 ± 0.25	0.97 ± 0.27	0.97 ± 0.25	1.21 ± 0.37	1.31 ± 0.28
	EOS (10^3^/*μ*L)	0.37 ± 0.23	0.31 ± 0.20	0.41 ± 0.30	0.30 ± 0.19	0.25 ± 0.18	0.35 ± 0.25

Data are expressed as means ± SD of ten animals in each group.

## Data Availability

The data used to support the findings of this study are available from the corresponding author upon reasonable request.
